# The Role of Adjuvant Acid Suppression on the Outcomes of Bleeding Esophageal Varices after Endoscopic Variceal Ligation

**DOI:** 10.1371/journal.pone.0169884

**Published:** 2017-01-24

**Authors:** Cheng-Kun Wu, Chih-Ming Liang, Chien-Ning Hsu, Tsung-Hsing Hung, Lan-Ting Yuan, Seng-Howe Nguang, Jiunn-Wei Wang, Kuo-Lun Tseng, Ming-Kun Ku, Shih-Cheng Yang, Wei-Chen Tai, Chih-Wei Shih, Pin-I Hsu, Deng-Chyang Wu, Seng-Kee Chuah

**Affiliations:** 1 Division of Hepato-gastroenterology; Department of Internal Medicine, Kaohsiung Chang Gung Memorial Hospital, Kaohsiung, Taiwan; 2 Department of Pharmacy, Kaohsiung Chang Gung Memorial Hospital, Kaohsiung, Taiwan; 3 School of Pharmacy, Kaohsiung Medical University, Kaohsiung, Taiwan; 4 Division of Hepato-gastroenterology; Department of Internal Medicine, Dalin Tzu Chi General Hospital, Buddhist Tzu Chi Medical Foundation, Chiayi, Taiwan; 5 Divisions of Gastroenterology, Yuan General Hospital, Kaohsiung, Taiwan; 6 Division of Gastroenterology; Pin-Tung Christian Hospital, Pin-Tung, Taiwan; 7 Division of Gastroenterology, Department of Internal Medicine, Kaohsiung Medical University Hospital and Kaohsiung Medical University, Kaohsiung, Taiwan; 8 Division of Gastroenterology; FooYin University Hospital, Pin-Tung, Taiwan; 9 Chang Gung University, College of Medicine, Kaohsiung, Taiwan; 10 Division of Hepato-gastroenterology; Department of Internal Medicine, ChiaYi Chang Gung Memorial Hospital, Chiayi, Taiwan; 11 Division of Gastroenterology, Department of Internal Medicine, Kaohsiung Veterans General Hospital, National Yang-Ming University, Kaohsiung, Taiwan; University Hospital Llandough, UNITED KINGDOM

## Abstract

The impact of adjuvant acid suppression via proton pump inhibitors or histamine-2 receptor antagonists after endoscopic variceal ligation remains uncertain. We therefore aimed to evaluate the effect of adjuvant acid suppression on the rebleeding and mortality rates in patients who received endoscopic variceal ligation and vasoconstrictor therapy for bleeding esophageal varices. Data from 1997 to 2011 were extracted from the National Health Insurance Research Database in Taiwan. A total of 1576 cirrhotic patients aged > 18 years with a primary diagnosis of acute esophageal variceal bleeding who received endoscopic variceal ligation therapy were screened. After strict exclusion, 637 patients were recruited. The exclusion criteria included patients with gastric variceal bleeding, failure in the control of bleeding, mortality within 12 hours, and history of hepatocellular carcinoma or gastric cancer. Patients were divided into two groups: the vasoconstrictors group (n = 126) and vasoconstrictors plus acid suppression group (n = 511). We observed that the rebleeding and mortality rates were not significantly different between 2 groups during hospitalization and the 15-year follow-up period after discharge. A Charlson score ≥3 (odds ratio: 2.42, 95% confidence interval: 1.55 ~3.79, P = 0.0001), presence of hepatitis C virus (odds ratio: 1.70, 95% confidence interval: 1.15 ~2.52, P = 0.0085), and cirrhosis (odds ratio: 1.69, 95% confidence interval: 1.08 ~2.66, P = 0.0229) were the independent risk factors of mortality after discharge. In conclusion, the results of the current study suggest that adjuvant acid suppression prescription to patients who received endoscopic variceal ligation and vasoconstrictor therapy for bleeding esophageal varices may not change the rebleeding and mortality outcomes compared to that for those who received endoscopic variceal ligation and vasoconstrictor agents without acid suppression.

## Introduction

Esophageal varices (EV) are one of the most common complications occurring in patients with cirrhosis. About one-third of patients with cirrhosis can experience their first episode of acute EV bleeding during follow-up, with a 70% recurrent bleeding rate and 20~50% mortality rate [1–3]. Fortunately, with recent advances in medicine and endoscopic hemostatic devices, a decrease in mortality rate has been observed over the past two decades [4–9].

Treatment for acute EV bleeding is now standardized, and includes endoscopic variceal ligation (EVL) combined with vasoconstrictor treatment and prophylactic antibiotics [2–4, 10–12]. However, the research regarding the role of an adjuvant proton pump inhibitor (PPI) in EV bleeding after EVL remains limited and unconvincing. Generally, for patients in the acute phase of cirrhosis with symptoms and signs of upper gastrointestinal bleeding, the use of PPIs before the diagnosis of EV bleeding is confirmed by endoscopy on arrival at the emergency room is common. Alaniz reported that 67~96.1% of patients with acute variceal bleeding received parenteral PPI therapy [13–14]. Moreover, esophageal ulcers are one of the medical events evoking caution after EVL. Patients with post-EVL esophageal ulcers may experience chest pain, odynophagia, and even bleeding from the ulcer itself [15]. Several studies have reported that PPI reduces post-EVL ulcer size through acid suppression [16–18]. Although Hidaka et al. reported that nearly half of arly half patients udy, Is on the outcomes of rebleeding and mortality is limited. ents might patients received long term PPI therapy with reduced treatment failures after EVL, a relationship between the use of PPI and the risk of postprocedural bleeding after prophylactic EVL was not demonstrated. Therefore, the impact of adjuvant PPI use on the outcomes for EVL in patients with EV bleeding, in terms of rebleeding and bleeding-related mortality, remains uncertain. Importantly, one may argue that the clinical importance of such findings is not known given the self-limiting nature of esophageal ulcers. In addition, accumulating data suggest that PPIs have other important negative effects on events in cirrhosis, such as spontaneous bacterial peritonitis and encephalopathy [19–21]. Thus, the use of PPI in acute EV bleeding and post-EVL care in Taiwan has been restricted by the National Health Insurance. Therefore, clinical physicians may sometimes prescribe histamine-2 receptor antagonists (H_2_RA) instead of PPI for acid suppression. Moreover, given that most experts and guidelines do not recommend the routine use of acid suppressive agents in acute variceal bleeding, further studies are needed to examine why this traditional and non-evidence-based orthodoxy, which appears to be prevalent in Taiwan and in many parts of the world, might be incorrect. Therefore, we conducted a 15-Year nationwide cohort study aimed to evaluate the effect of adjuvant acid suppression (PPI and H_2_RA) in patients who received EVL and vasoconstrictor therapy for EV bleeding on rebleeding and mortality, compared to those who received the standard treatment for EVL and vasoconstrictor therapy without acid suppression.

## Materials and Methods

### Ethical approval and informed consent statement

The study protocol was approved by the institutional review board and the Ethics Committee of Chang Gung Memorial Hospital and Kaohsiung Medical University Hospital, Kaohsiung, Taiwan (IRB104-5851B). The Ethics Committee waived the requirement for informed consent for this study, and all the data were analyzed anonymously. The methods were carried out in accordance with the relevant guidelines and regulations

### Data source

Taiwan’s National Health Insurance program was initiated in 1995 and covers 99% of Taiwan’s 23 million individuals. In 1999, the Bureau of National Health Insurance began to release all claims data in electronic format to the public under the National Health Insurance Research Database (NHIRD) project [22]. As yet, the NHIRD has been extensively used in numerous epidemiological studies. In the present study, a total of 1,000,000 individuals (approximately 5% of Taiwan’s population) were randomly selected from Taiwan’s NHIRD for analysis. Claims data were collected from Taiwan’s NHIRD at the National Health Research Institutes. The data analysts were staff members of the Kaohsiung Medical Center, a site of the Collaboration Center of Health Information Application, Ministry of Health and Welfare. For research purposes, the institute released a cohort dataset of one million randomly selected individuals and a dataset of patients with a catastrophic illness who were alive in 2011. The institute collected all records for these individuals from 1997 to 2011. Comprehensive health care data included the enrolment files, claims data, catastrophic illness files, and a registry for drug prescriptions. The 9th Revision codes were used to define diseases. In the cohort dataset, each patient’s original identification number was encrypted for privacy.

### Study groups, inclusion and exclusion criteria

**Fig 1:**
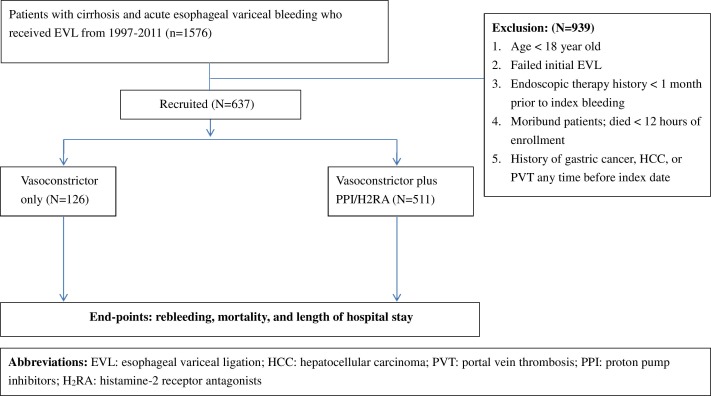
Schematic flowchart of the study design.

Fig 1 shows a schematic flowchart of the study design. The inclusion criteria consisted of patients with cirrhosis aged ≥ 18 years at admission and a primary discharge diagnosis code of EV bleeding as according to the International Classification of Diseases 9th revision [International Classification of Diseases, Ninth Revision, Clinical Modification (ICD-9-CM) codes: 456.0, 456.1, 456.2 and 530.82]. The code 456.1 was only included when the patients had concomitant EVL during the index hospitalization. The ICD-9-CM code for cirrhosis is 571, which was defined as 1 year prior to the index bleeding. We recruited patients who received EVL with ICD-9-CM codes of 42.91 but excluded those with ICD-9-CM codes of 444.3 (endoscopic control of gastric or duodenal bleeding). A total of 1576 patients with cirrhosis and EV bleeding were identified. The following patients were excluded: patients with gastric variceal bleeding (ICD-9-CM code: 456.8), patients with a failure to control the bleeding via emergent endoscopic treatments, patients with a history of endoscopy therapy (including sclerotherapy or ligation) less than one month prior to the index bleeding, moribund patients who died within 12 hours of enrollment (death event occurring in patients with a length of hospital stay less than 1 day), patients with a history of hepatocellular carcinoma or portal vein thrombosis (ICD-9-CM code: 155, 452) found at any time before the index date, and patients with a history of gastric cancer (ICD-9-CM code: 151) any time before index date. In addition, some esophageal varices might have originated from pre-hepatic portal hypertension caused by cholangiocarcinoma and pancreas cancer. In the current study, we have focused only on patients with acute esophageal variceal bleeding caused by liver cirrhosis.

Importantly, the aim of this study was to assess the impact of adjuvant acid suppression prescription on the outcome in patients who received EVL and vasoconstrictor therapy for bleeding EVs compared to those who did not received adjuvant acid suppression. Therefore, we strictly enrolled only patients with a concomitant ICD code of EVL (ICD-9-CM code: 42.91) and the prescription of consecutive doses of vasoconstrictor-based therapy such as terlipressin (Anatomical Therapeutic Chemical and Defined Daily Dose (ATC-DDD) Index: H01BA04), somatostatin (ATC-DDD Index: H01CB01), octreotide (ATC-DDD Index: H01CB02), and prophylactic antibiotics with or without acid suppression (ATC-DDD Index: PPI, A02BC; H_2_RA, A02BA). In addition, we enrolled only patients who received consecutive doses of acid suppression before the index endoscopy or during the index hospitalization. Patients receiving only a single dose were not included.

Finally, a total of 637 patients with cirrhosis and EV bleeding who received EVL and vasoconstrictor-based therapy during hospitalization were recruited for analysis. These patients were divided into two groups: those who received EVL and vasoconstrictor treatment only (N = 126), and those who received an adjuvant acid suppression prescription prior to EVL and vasoconstrictor treatment (N = 511). A total of 457 patients received vasopressor and PPI treatment, while 54 patients received vasopressor and H_2_RA treatment. We initially performed an analysis using 3 groups (vasopressor only, vasopressor plus PPI, vasopressor plus H_2_RA), but did not find any significant differences in rebleeding and mortality among the 3 groups. However, these data were somewhat biased by the relatively few numbers of patients receiving H_2_RA. Therefore, we decided to combine patients receiving PPI and H_2_RA into an “acid suppression” group.

### Definition of variables

Demographic information including age and sex was obtained. Comorbid conditions were recorded if listed among the diagnoses for the hospitalization. The burden of comorbid illness was assessed based on the Deyo modification of the Charlson comorbidity index (CCI). The CCI ranges from 0 to 17, with higher numbers representing a greater comorbidity burden. The CCI incorporates 17 comorbid conditions and has been shown to be a well-validated measure of comorbidity adjusting for disease burden in administrative data [23]. Comorbidities, including previous congestive heart failure, cerebral vascular disease, diabetes, hypertension, chronic obstructive pulmonary disease, and chronic kidney disease were defined as diseases diagnosed on admissions before the index hospitalization and during the follow-up period. Patients with a diagnosis of hepatorenal syndrome (ICD-9-CM code: 572.4), ascites (ICD-9-CM code: 789.5), variceal hemorrhage (ICD-9-CM code: 456.0, 456.20), hepatic encephalopathy (ICD-9-CM code: 572.2), or jaundice (ICD-9-CM code: 782.4) were defined as having decompensated cirrhosis. Accordingly, patients with cirrhosis without any of the above ICD-9-CM codes were considered to have compensated liver cirrhosis. Rebleeding was defined as repeated endoscopic therapy (ICD-9-CM code: 43043B), use of a Sengstaken–Blakemore tube (ICD-9-CM code: 96.06), or surgery within the same admission.

### Outcomes

The primary outcomes included rebleeding and death events during the index hospitalization and after discharge from the index hospitalization. The secondary outcome consisted of the length of hospital stay for the index hospitalization.

### Statistics

Continuous data are presented as means ± standard deviation (SD) and categorical data are presented as frequencies and percentages. Pearson’s chi-square or Fisher’s exact 2-tailed tests were used for the analysis of categorical data, while continuous variables were analyzed using the t-test, where appropriate. A multiple logistic regression analysis was performed to examine the factors associated with treatment allocation. Kaplan-Meier survival curves and Cox regression were used to compare the outcomes of interest between groups. Adjustments were made in the regression analysis for patient demographics, clinical conditions, and medication usage. Two-tailed p-values < 0.05 were considered statistically significant. All statistical analyses were conducted using SAS version 9.3 (SAS Institutes Inc., Cary, NC, USA, 2013).

## Results

### Demographic data and outcomes

Demographic data for the two groups are shown in Table 1. The two groups had a similar age distribution (age [mean ± SD]: vasoconstrictors only, 58.94±16.57 years; vasoconstrictors plus acid suppression, 58.84±16.97 years; P = 0.9540). A non-significant group difference was also observed when age intervals were established for further comparison. In addition, the groups did not significantly differ in CCI (1.46±1.90 vs. 2.50±1.18±1.71, respectively; p = 0.1077). There were 34 cancer patients; these patients had tongue cancer, nasopharyngeal cancer, hypopharyngeal cancer, cervical cancer, breast cancer, vaginal cancer, or lung cancer. None of these patients died after a bleeding event during the 15 years of follow-up.

**Table 1:** Baseline Characteristics.

Characteristics	Vasoconstrictors only(n = 126)		Vasoconstrictors plus PPI/H_2_RA (n = 511)		p-value
	N	%	N	%	
**Age, years (mean ± SD)**	58.94±16.57		58.84±16.97		0.9540
18–29	2	1.59%	16	3.13%	0.8462
30–39	14	11.11%	43	8.41%	
40–49	29	23.02%	120	23.48%	
50–59	23	18.25%	91	17.81%	
60–69	18	14.29%	84	16.44%	
≥ 70	40	31.75%	157	30.72%	
**Sex**					
Female	20	15.87%	146	28.57%	0.0036
Male	106	84.13%	365	71.43%	
**Etiology**					
HBV	40	31.75%	104	20.35%	0.0062
HCV	30	23.81%	83	16.24%	0.0464
Other viral hepatitis	0	0.00%	0	0.00%	—
Alcoholism	49	38.89%	162	31.70%	0.1248
**Charlson score**					
0	60	47.62%	273	53.42%	0.3483
1	16	12.70%	77	15.07%	
2	20	15.87%	70	13.70%	
≥ 3	30	23.81%	91	17.81%	
**Charlson score (mean ± SD)**	1.46±1.90		1.18±1.71		0.1077
**Charlson Comorbidity**					
Acute myocardial infarction	0	0.00%	2	0.39%	0.4818
Congestive heart failure	2	1.59%	7	1.37%	0.8531
Peripheral vascular disease	0	0.00%	4	0.78%	0.3191
Cerebral vascular accident	2	1.59%	20	3.91%	0.2002
Dementia	1	0.79%	2	0.39%	0.5547
Pulmonary disease	6	4.76%	32	6.26%	0.5242
Connective tissue disorder	0	0.00%	1	0.20%	0.6192
Peptic ulcer	29	23.02%	115	22.50%	0.9023
Liver disease	59	46.83%	186	36.40%	0.0312
Diabetes	18	14.29%	74	14.48%	0.9554
Diabetes complications	5	3.97%	21	4.11%	0.9428
Paraplegia	0	0.00%	1	0.20%	0.6100
Renal disease	3	2.38%	12	2.35%	0.9827
Cancer	12	9.52%	22	4.31%	0.0196
Severe liver disease	9	7.14%	15	2.94%	0.0263
HIV	0	0.00%	0	0.00%	—
**Cirrhosis**	104	82.54%	371	72.60%	0.0218
Compensated cirrhosis	104	82.54%	370	72.41%	0.0196
Decompensated cirrhosis	52	41.27%	174	34.05%	0.1293

Abbreviations: PPI: proton pump inhibitors; H_2_RA: histamine-2 receptor antagonists; SD: standard deviation;HBV: hepatitis B virus; HCV: hepatitis C virus; HIV, human immunodeficiency virus;EVB: esophageal variceal bleeding; NHRID: National Health Research Institute database

The outcome data are shown in Table 2. During hospitalization, the rebleeding rate did not significantly differ between the 2 groups (15.08% vs. 12.13%, respectively; P = 0.3739) regardless of repeat endoscopy, surgery, or the use of a Sengstaken–Blakemore tube. In addition, the groups did not differ in the length of hospital stay (9.27±10.51 days versus 9.04±9.02 days, P = 0.8072). No death events occurred during hospitalization in either group.

**Table 2:** Outcomes of the study population.

Characteristics	Vasoconstrictors only (n = 126)		Vasoconstrictors plus PPI/H_2_RA (n = 511)		p-value
	N	%	N	%	
**Adverse outcome (During index History)**					
Rebleeding	19	15.08%	62	12.13%	0.3739
Repeat endoscopy	1	0.79%	1	0.20%	0.2826
Surgery	3	2.38%	14	2.74%	0.8229
SB tube	16	12.70%	49	9.59%	0.3017
Death	0	0.00%	0	0.00%	—
**Length of Stay (d)**					
Mean±SD	9.27±10.51		9.04±9.02		0.8072
Median (range)	6 (1–63)		6 (1–86)		0.1101
**Adverse outcome (Discharge after index History)**	**Vasoconstrictors only (n = 126)**		**Vasoconstrictors plus PPI/H**_2_**RA (n = 511)**		
Rebleeding	5	3.97%	17	3.33%	0.7240
Death	53	42.06%	206	40.31%	0.7201

Abbreviations: PPI: proton pump inhibitors; H_2_RA: histamine-2 receptor antagonists; SB: Sengstaken–Blakemore

**Fig 2:**
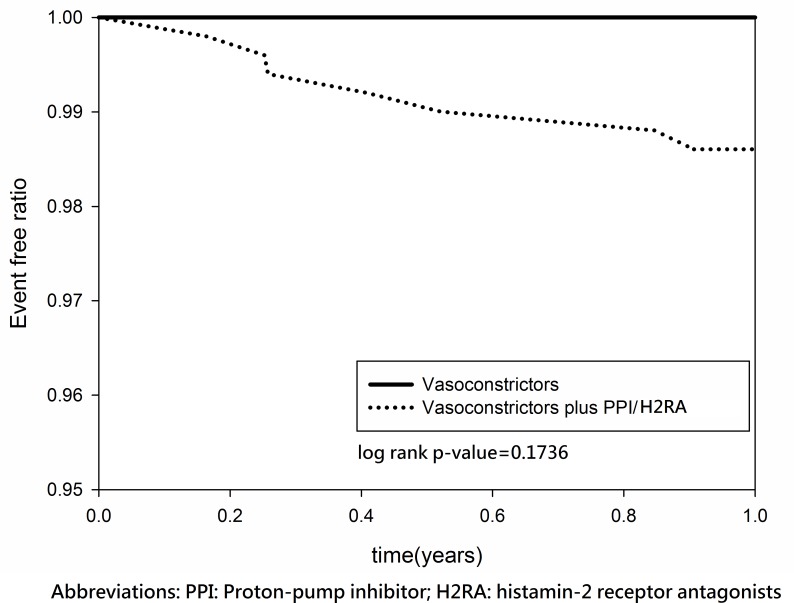
Kaplan–Meier estimates for the absence of re-bleeding in patients with cirrhosis and esophageal variceal bleeding (1 year follow-up).

**Fig 3:**
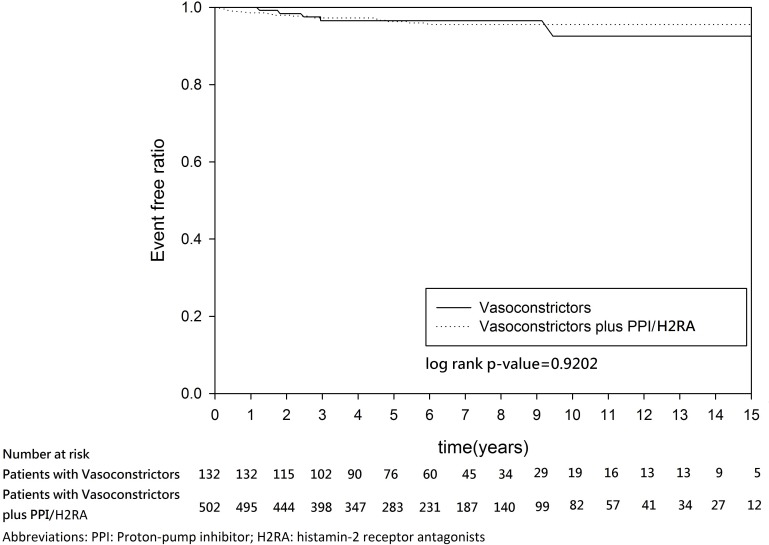
Kaplan–Meier estimates for the absence of re-bleeding in patients with cirrhosis and esophageal variceal bleeding (15 years follow-up).

**Fig 4:**
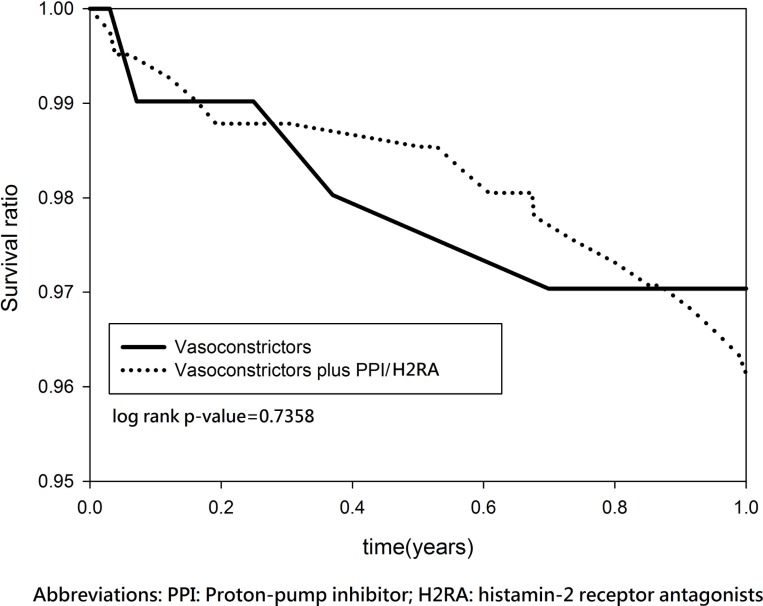
Kaplan–Meier estimates for survival in patients with cirrhosis and esophageal variceal bleeding (1 year follow-up).

**Fig 5:**
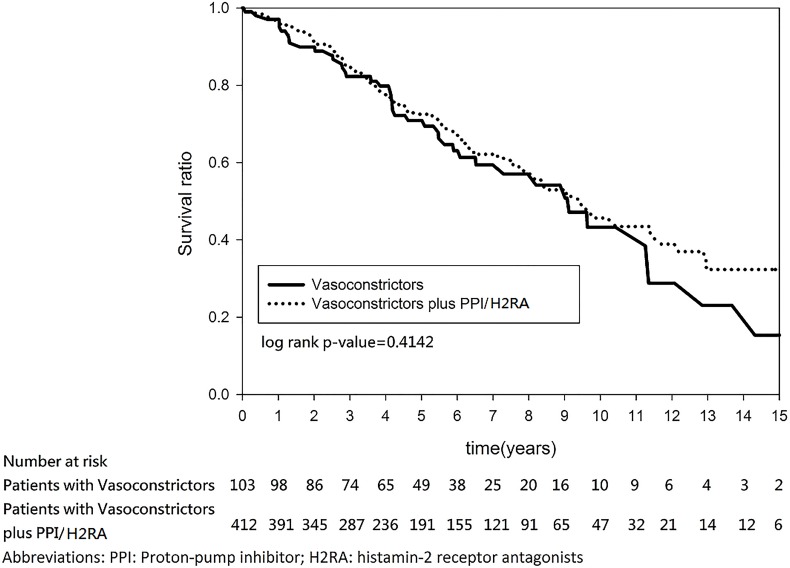
Kaplan–Meier estimates of survival in patients with cirrhosis and esophageal variceal bleeding (15 years follow-up).

After discharge, the two groups had a similar rebleeding rate (3.97% versus 3.33%, P = 0.7240) and mortality rate (42.06% versus 40.31%, P = 0.7201). The median/mean follow-up duration was 9.126/8.5265 years. In the log-rank test and Kaplan–Meier survival analysis, no significant group differences in the 1-year rebleeding rate (log-rank P = 0.1736) (Fig 2) or the 15-year rebleeding rate (log-rank P = 0.9202) (Fig 3) were found. Similarly, no significant group differences in 1-year mortality (log-rank P = 0.7358) (Fig 4) or 15-year mortality (log-rank P = 0.4142) (Fig 5) were found.

### Independent risk factors for rebleeding in all study populations during index hospitalization

No significant independent risk factors for rebleeding were identified with respect to age, sex, CCI, cirrhosis, and etiology (including hepatitis B virus, hepatitis C virus, and alcoholism) **(Table 3)**. In addition, there was no significant difference in rebleeding when patients with vasoconstrictors plus acid suppression medications were compared to those with vasoconstrictors only after EVL (odds ratio: 0.78, 95% CI: 0.44~1.39, P = 0.4052).

**Table 3:** Independent risk factors of rebleeding in all study populations during the index hospitalization.

Variable	Multivariate analysis			
	OR	95% CI		p-value
**Group**				
Vasoconstrictors	1.00			
Vasoconstrictors plus PPI/**H**_2_**RA**	0.78	0.44	1.39	0.4052
**Age**				
18–29	1.00			
30–39	0.36	0.07	1.80	0.2130
40–49	0.70	0.18	2.70	0.6092
50–59	0.69	0.18	2.72	0.5968
60–69	0.92	0.23	3.61	0.9041
≥ 70	0.80	0.21	3.01	0.7405
**Sex (male is reference)**	0.78	0.43	1.42	0.4208
**Charlson score**				
0	1.00			
1	1.16	0.59	2.31	0.6667
2	1.19	0.59	2.38	0.6310
≥ 3	0.64	0.30	1.34	0.2336
**Cirrhosis**	1.02	0.51	2.05	0.9523
**HBV**	1.26	0.71	2.22	0.4376
**HCV**	0.96	0.50	1.85	0.8976
**Alcoholism**	1.08	0.62	1.89	0.7788

Abbreviations: CI: confidence interval; PPI: proton pump inhibitors; H_2_RA: histamine-2 receptor antagonists; HBV: hepatitis B virus; HCV: hepatitis C virus

### Independent risk factors for rebleeding and death in all study populations during the 15-year follow-up period

In the multivariate analysis, no significant independent risk factors for rebleeding after discharge were identified with respect to age, sex, CCI, cirrhosis, and etiology (including hepatitis B virus, hepatitis C virus, and alcoholism). In addition, there was no significant difference in rebleeding after discharge when patients with vasoconstrictors plus acid suppression medications after EVL were compared to those with vasoconstrictors only after EVL (odds ratio: 0.77, 95% CI: 0.28~2.14, P = 0.6145) (Table 4).

**Table 4:** Independent risk factors of rebleeding in all study populations during the 15-year follow-up period.

Variable	Multivariate analysis			
	HR	95% CI		p-value
**Group**				
Vasoconstrictors	1.00			
Vasoconstrictors plus PPI/**H**_2_**RA**	0.77	0.28	2.14	0.6145
**Age**	1.02	0.99	1.05	0.1651
**Sex (male is reference)**	1.59	0.64	3.96	0.3179
**Charlson score**				
0	1.00			
1	1.19	0.29	4.83	0.8130
2	2.30	0.72	7.36	0.1599
≥ 3	1.48	0.42	5.21	0.5423
**Cirrhosis**	1.08	0.30	3.90	0.9012
**HBV**	0.54	0.15	1.90	0.3361
**HCV**	1.82	0.65	5.15	0.2563
**Alcoholism**	1.06	0.39	2.89	0.9116

Abbreviations: CI: confidence interval; PPI: proton pump inhibitors; H_2_RA: histamine-2 receptor antagonists; HBV: hepatitis B virus; HCV: hepatitis C virus

Significant independent risk factors of mortality after discharge identified in the multivariate analysis included a high CCI score (≥3; odds ratio: 2.42, 95% CI: 1.55 ~3.79, P = 0.0001), presence of hepatitis C virus (odds ratio: 1.70, 95% CI: 1.15 ~2.52, P = 0.0085), and cirrhosis (odds ratio: 1.69, 95% CI: 1.08 ~2.66, P = 0.0229) (Table 5). However, there was no significant difference in mortality after discharge when patients with vasoconstrictors plus acid suppression medications after EVL were compared to those with vasoconstrictors alone after EVL (odds ratio: 0.99, 95% CI: 0.70~1.40, P = 0.9446).

**Table 5:** Independent risk factors of death in all study populations during 15-year follow-up period.

Variable	Multivariate analysis			
	HR	95% CI		p-value
**Group**				
Vasoconstrictors	1.00			
Vasoconstrictors plus PPI/**H**_2_**RA**	0.99	0.70	1.40	0.9446
**Age**	1.01	1.00	1.01	0.3134
**Sex (male is reference)**	1.17	0.84	1.64	0.3490
**Charlson score**				
0	1.00			
1	1.17	0.73	1.89	0.5197
2	1.18	0.71	1.96	0.5353
≥ 3	2.42	1.55	3.79	0.0001
**Cirrhosis**	1.69	1.08	2.66	0.0229
**HBV**	1.05	0.70	1.57	0.8101
**HCV**	1.70	1.15	2.52	0.0085*
**Alcoholism**	1.04	0.72	1.51	0.8203

Abbreviations: CI: confidence interval; PPI: proton pump inhibitors; H_2_RA: histamine-2 receptor antagonists; HBV: hepatitis B virus; HCV: hepatitis C virus

## Discussion

Although the effective impact of PPI as a strong antacid in the treatment of peptic ulcer diseases via inhibition of the final step in gastric acid production is well established, the impact of PPI or H_2_RA on the outcomes in gastroesophageal varices bleeding remains inconclusive [24]. Post-procedure esophageal ulcer bleeding is a rare, but severe complication [25]. As mentioned earlier, the use of PPIs has been restricted by the National Health Insurance in Taiwan; as a result, many clinical physicians may prescribe H_2_RA instead of PPI for the treatment of bleeding EVs after EVL as an adjuvant to vasoactive agents. In the current study, the use of acid suppression was quite prevalent (80.2%, 511/637) in patients with cirrhosis and EV bleeding. The results of the current 15-year nationwide cohort study demonstrated that the outcomes, in terms of rebleeding and mortality, among cirrhotic patients who received EVL for bleeding varices were similar between those who received adjuvant prescriptions of acid suppression in addition to vasoactive agents, and those who received the standard treatment for EVL, which consists of vasoconstrictor therapy without acid suppression.

Among studies evaluating post-EVL complications, Shaheen et al. reported that endoscopy 10–14 days post-banding revealed significantly smaller ulcers for patients who were prescribed with PPI in a double-blind randomized controlled trial [16]. Similarly, Young et al. showed that post-EVL ulcers were shallower and healed more quickly compared to ulcers caused by endoscopic injection therapy [25]. Nijhawan et al. observed that 60% of post-EVL ulcers were healed within 2 weeks and 100% were healed within 3 weeks [26]. Although numerous studies demonstrate that PPIs effectively reduce post-procedure ulcer size and accelerate ulcer healing, none have demonstrated altered rebleeding or mortality rates in patients with post-treatment EV bleeding [15–17, 27]. The explanation provided was that most rebleeding events arise from varices rather than ulcers, and therefore the value of PPI use was questioned.

Vanbiervliet et al. reported that 21 of 605 patients (3.4%) with bleeding varices experienced post-banding ulcer bleeding [28]. PPI was proposed to improve ulcer healing, and hence decrease the rebleeding rate of EV bleeding. In an interesting randomized controlled study that directly compared PPIs with vasoconstrictors in patients with cirrhosis and EV bleeding successfully treated by EVL, Lo et al. observed that the effect of adjuvant therapy with PPI infusion was similar to its combination with vasoconstrictor infusion in terms of the initial hemostasis and very early rebleeding rate, and was associated with fewer adverse events [18]. The authors hypothesized that the impact of the vasoconstrictor was diminished because the portal pressure was not elevated, and therefore PPIs should be used to prevent ulcer bleeding. However, in the current study, there was no difference in mortality between the two groups. Moreover, the long-term use of PPI raises concerns regarding adverse effects such as *Clostridium difficile* infection, spontaneous bacterial peritonitis, bone fractures, pneumonia, etc. [29–31]. In a systemic review conducted by Lo et al. [21], the best evidence for PPIs was suggested to still be lacking, and the use of a short-course (10 days) of PPIs to reduce ulcer size whenever ulcer healing was a concern was supported. Lodato et al. further suggested that the use of PPI with regard to the prevention and treatment of esophageal complications after banding or sclerotherapy of esophageal varices may be more of a tradition rather than an evidence-based practice, due to the lack of convincing evidence [32].

In the present study, no significant differences in rebleeding or mortality during index hospitalization and 15-year follow-up were found between the two groups. A high CCI, presence of hepatitis C virus, and cirrhosis were found to be significant independent risk factors of mortality, consistent with a previous study [33]. This result appears rational as comorbidities have always been important prognostic factors for patients with cirrhosis. Importantly, the implication of the present results is that the adjuvant use of acid suppression does not alter the outcomes.

There are several limitations in this study. First, the study used a retrospective cohort design with observations based on hospitalized patients with EV bleeding. The diagnosis of EV bleeding and identification of comorbidities are dependent on the accuracy of the coding procedures. Certain selection biases may exist, and caution must be taken in extrapolating the results. A second limitation concerns the heterogeneous nature of the acid suppressive drug group, which included PPI and H2RA. Although the use of 'adjuvant' acid suppression might influence early rebleeding or mortality, many other variables exist, including continued acid suppressive therapy, which may bias the longer-term data. In addition, a wide variety of drugs and regimens may have also contributed to the presented long-term outcome data. Thus, post-discharge acid suppression and other confounding factors must be considered. Moreover, information was not available regarding lifestyle factors (e.g., alcohol consumption, smoking, and malnutrition), which are reported to be prognostic factors in patients with cirrhosis [34, 35]. These factors could have confounded our results if they were more prevalent in one group of patients. Third, data regarding the use of beta-blockers and antibiotic prophylaxis was lacking. Although it has not yet been standardized, such use could affect the final results of survival. Lastly, although a previous Taiwanese report has suggested that the long-term use of PPIs is associated with important negative effects on events in cirrhosis, such as encephalopathy, no data were available in current study to clarify such effects for short-term PPI use [19]. In spite of these limitations, our study has strengths and clinical implications. Use of data from a nationwide database minimizes the potential for biases that may be seen in single-center studies. This database serves as a powerful tool to study the impact of acid suppression on the outcomes of patients with cirrhosis and EV bleeding, especially after EVL.

In conclusion, the results of the current study suggest that adjuvant acid suppression prescription to patients who received EVL and vasoconstrictor therapy for bleeding EVs may not change the rebleeding and mortality outcomes compared to those for patients who received EVL and vasoconstrictor agents without acid suppression.

## Supporting Information

S1 Dataset This file provides the data of yearly death (years 1–15) & rebleed p-values. (XLS)

## Abbreviations

EV: esophageal variceal
EVL: endoscopic variceal ligation
NHRID: National Health Research Institute database
PPI: proton pump inhibitors
H_2_RA: histamine-2 receptor antagonists
SD: standard deviation
HBV: hepatitis B virus
HCV: hepatitis C virus
